# Applying DNA rolling circle amplification in fluorescence imaging of cell surface glycans labeled by a metabolic method†
†Electronic supplementary information (ESI) available. See DOI: 10.1039/c6sc02089e


**DOI:** 10.1039/c6sc02089e

**Published:** 2016-06-14

**Authors:** Xiaoru Zhang, Ruijuan Li, Yuanyuan Chen, Shusheng Zhang, Wenshuang Wang, Fuchuan Li

**Affiliations:** a Key Laboratory of Sensor Analysis of Tumor Marker , Ministry of Education , College of Chemistry and Molecular Engineering , Qingdao University of Science and Technology , Qingdao 266042 , P. R. China; b Shandong Province Key Laboratory of Detection Technology for Tumor Makers , College of Chemistry and Chemical Engineering , Linyi University , Linyi 276000 , P. R. China . Email: shushzhang@126.com; c National Glycoengineering Research Center and State Key Laboratory of Microbial Technology , Shandong University , Jinan 250100 , P. R. China . Email: fuchuanli@sdu.edu.cn

## Abstract

Glycans on the cell surfaces are essential for cellular communication. Metabolically labeling glycans can introduce unnatural sugars into cellular glycans, which can facilitate further labeling. We report herein imaging cell surface glycosylation by using click chemistry and DNA rolling circle amplification (RCA) to improve detection sensitivity. Through the RCA amplification, the image resolution of a cell was significantly improved and much fewer unnatural sugars were used than required previously. The advantage of this method is that it avoids introducing too much unnatural sugar, which can interfere with normal, physiological cell function. Simultaneously, the enhanced fluorescence intensity conveniently facilitates the detection of cells' own biosynthetic glycans by simply using a microplate reader. The results indicate that the metabolically labelling ability is different for different carbohydrates and different cells. Next, the RCA technique was adopted in a fluorescence resonance energy transfer (FRET)-based methodology that facilitated the glycan imaging of specific proteins on the cell surface. This method is broadly applicable to imaging the glycosylation of cellular proteins. Our results highlight the applications of RCA in metabolic glycan labeling, which can be used to monitor the glycosylation status on cells, and study the means by which glycosylation regulates cell function.

## Abstract 2

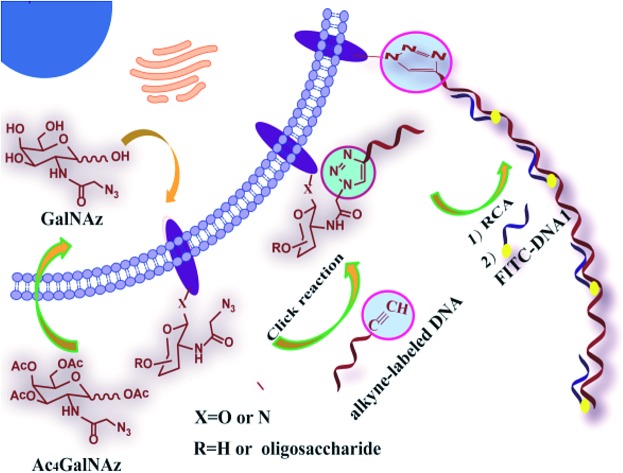
We report herein imaging cell surface glycosylation by using click chemistry and DNA rolling circle amplification (RCA) to improve detection sensitivity..

## Introduction

Protein glycosylation is an important post-translational modification. Aberrant alterations in N- and O-linked glycans are involved in many diseases.^1^,^2^ Consequently, research on glycan structure and function, particularly visualization of glycans on the cell surface, is critical for elucidating the biological role of protein glycosylation in the dynamic environment of cells. However, glycosylation is a complex process due to its intrinsic heterogeneity.^3^ Glycans cannot be visualized alone due to a lack of chromophores or fluorophores. Moreover, unlike proteins or nucleic acids, it is difficult for glycans to be detected using specific antibodies or complementary sequences.^4^,^5^ Although, there are some reports on the evaluation of cell surface N-glycan expression based on the recognition of lectin,^6^,^7^ this method is unsuitable for analyzing O-linked glycans because their biosynthesis is complex and requires extensive prior digestion with glycosidases.^8^ Thus, glycan detection remains the main obstacle in glycosylation studies.

There are several publications of detecting protein glycosylation in cells using *in situ* proximity ligation assay.^9^–^11^ These methods offer new opportunities for glycopeptide identification, provided that antibodies for both protein and carbohydrate are available.^9^ But as we know, it is difficult to obtain antibodies against glycans, and their binding constants are rather low. In fact, so far very few anti-glycan antibodies are commercially available. Even for lectins, it is difficult for antibodies to be widely used due to their low availability and weak binding capacity. This is a bottleneck for investigating the process of protein glycosylation. Recently, a metabolic glycan labeling technique has been used to hijack a cell's biosynthetic process. This was first reported by Reutter's group^12^ and then extended by Bertozzi's group.^13^ This strategy has been used to visualize glycans on cells,^14^–^17^ virus^18^ and living organisms, such as zebrafish,^19^–^21^ *Caenorhabditis elegans*^22^ and mice.^3^,^23^ Carbohydrates functionalized with azide groups can be incorporated into the glycans of an organism *via* the cell's own metabolic machinery. Next, the azide group assembled in the glycan facilitates further coupling with a second bioorthogonal reagent through a covalent bond. Azide groups can directly react with alkyne-functionalized dyes^15^,^16^,^24^–^26^ or with labeled antibodies and lectins through two sequential bioorthogonal reactions.^3^,^23^ In the latter case, the antibodies or lectins can be labeled with more than one fluorophore, and the fluorescence intensity is enhanced accordingly. However, this enhancement is restricted due to the limited number of fluorophores conjugated to antibodies or lectins. On the other hand, carbohydrate metabolism can change all corresponding carbohydrates on a cell surface to the unnatural sugars without discrimination. If the concentration of added unnatural sugar is too high, the normal physiological function of a cell can be interfered with.^27^ Therefore, the methods for sensitive visualization of glycans on a cell surface by using as few azido-sugars as possible would be greatly helpful for understanding the glycosylation process.

Rolling circle amplification (RCA) is an isothermal DNA replication technique, which can generate a long single DNA strand with multiple repeat units.^28^–^30^ Recently, RCA has been used in fluorescence *in situ* imaging of microRNA in tumor cells,^31^–^33^ and tumor-specific delivery of drugs.^34^ However, until now, there has no report on the fluorescence imaging of carbohydrates on a cell surface using the RCA method. Here, we applied nucleic acid technology to glycochemistry, which could amplify the detection signal of a carbohydrate through nucleic acid amplification. Alkyne-functionalized DNA reacts with an azide group on a cell-surface glycan through click chemistry. The DNA assembled on the cell surface can further produce a long single DNA strand after RCA. Since exponential amplification by RCA allows to detect even a single DNA or RNA molecule,^31^ we hypothesized that this strategy can be used to investigate the subtle progress of glycosylation with more sensitivity. In addition, due to the lack of selectivity of the metabolic glycan labeling method, which leads to labeling of all glycoproteins and glycolipids containing the added unnatural sugar,^27^,^35^,^36^ we further utilized fluorescence resonance energy transfer (FRET) to specifically image glycoform-bearing proteins of interest.

## Results and discussion

### Cytotoxicity tests of azido-sugars in different doses

**Fig. 1:**
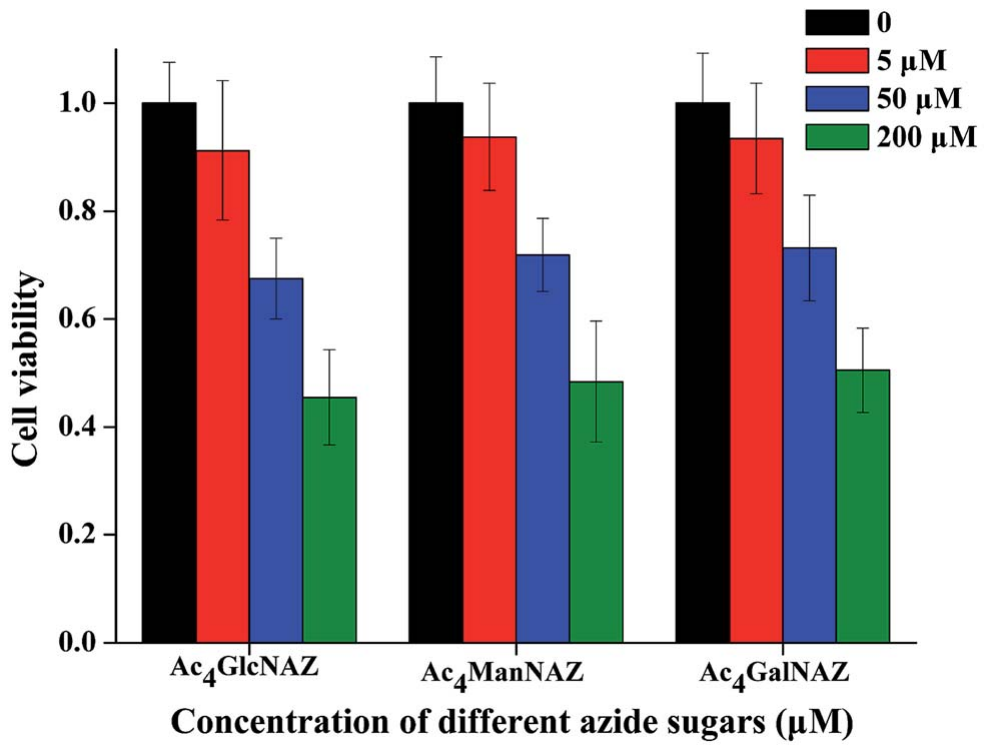
Growth and proliferation rate of B16 cells treated with different doses of three different unnatural sugars for 3 days. The data represent means ± s.d. (*n* = 3).

Studies have reported that per-*O*-acetylated azido sugars such as Ac_4_GalNAz can diffuse into the cells passively and are deacetylated under the action of intracellular esterases. The resulting deacetylated azido-sugars are converted to their high-energy donor forms (nucleotide sugars). These nucleotide sugars such as UDP-GalNAz are then directly exploited in glycan synthesis or are converted to other nucleotide sugars before being integrated into newly synthesized glycans. For example, UDP-GalNAz can be epimerized to UDP-GlcNAz by UDP-galactose 4′-epimerase.^15^ As mentioned above, various fluorescence imaging technologies have been developed for the detection of glycan biosynthesis using the azido-sugar metabolic labeling method.^23^–^26^ However, in these strategies, the consumption of azido-sugars is considerably high to produce clear images. For example, 500 μM,^27^ 100 μM,^35^ and 50 μM^15^ azido-sugars have been used previously. The introduction of high concentrations of unnatural azido-sugars to the cell may interfere with the normal physiological processes of the cell. To test this viewpoint, here we test the effect of azido-sugar concentration on the growth of B16 cells. From the results shown in Fig. 1 we can see that in all three tests, per-*O*-acetylated azido-sugars (Ac_4_ManNAz, Ac_4_GlcNAz and Ac_4_GalNAz) show significantly dose-dependent inhibition to the proliferation of B16 cells. Growth and proliferation rate of B16 cells can be affected significantly after treatment with 50 and 200 μM azido-sugar, while treatment with 5 μM azido-sugar has much less impact on the cell viability. In fact, similar results were also found in a previous study, in which Ac_4_ManNAz dose-dependently inhibited the growth of HeLa cells.^23^ To decrease the adverse effects, it is essential to reduce the dose of azido-sugar as much as possible by increasing the sensitivity of the detection method. In this study, the RCA technique was adopted to solve this problem.

### Design for RCA-assisted metabolic labeling of cell surface glycans

**Scheme 1:**
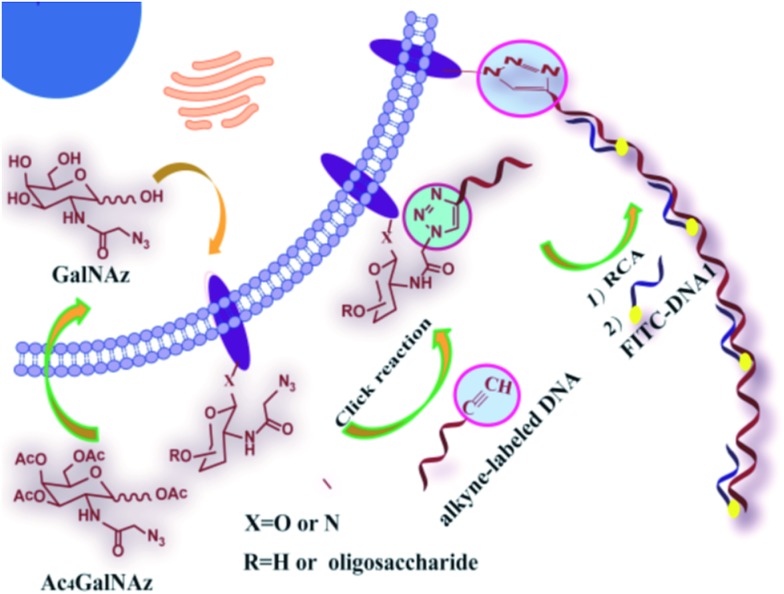
General strategy for DNA RCA-assisted metabolic labeling of cell surface glycans.

As shown in Scheme 1, the azide group of azido-sugars integrated into glycans was covalently coupled with alkyne-functionalized DNA, which facilitated the introduction of DNA on the cell surface. The assembled DNA could initiate an *in situ* RCA reaction in the presence of phi29 DNA polymerase and dNTPs, generating a long tandem repeated sequence. The hybridization of the RCA product with high quantities of FITC-modified detection probe can produce a super-bright fluorescence image under a fluorescence microscope, and thus the proposed method can provide information about the localization and distributions of carbohydrates on the cell surface by using relatively low concentration of azido-sugar.

### The amplification effect of RCA for glycan metabolic labeling

**Fig. 2:**
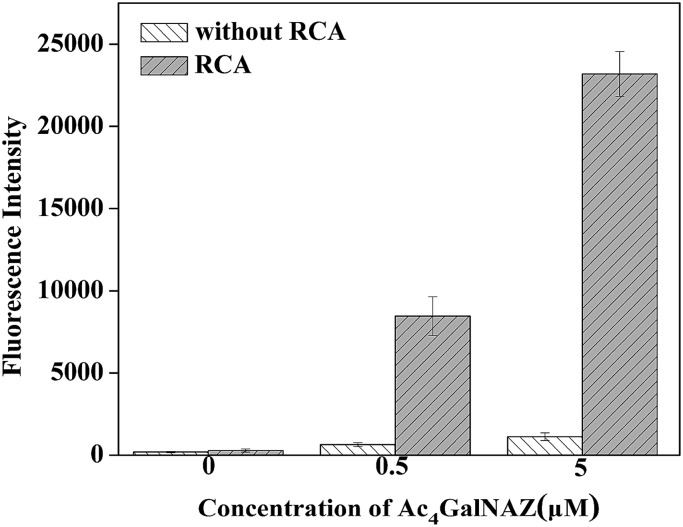
Fluorescence detection for 4TO7 cells treated with different concentrations of Ac_4_GalNAz.

**Fig. 3:**
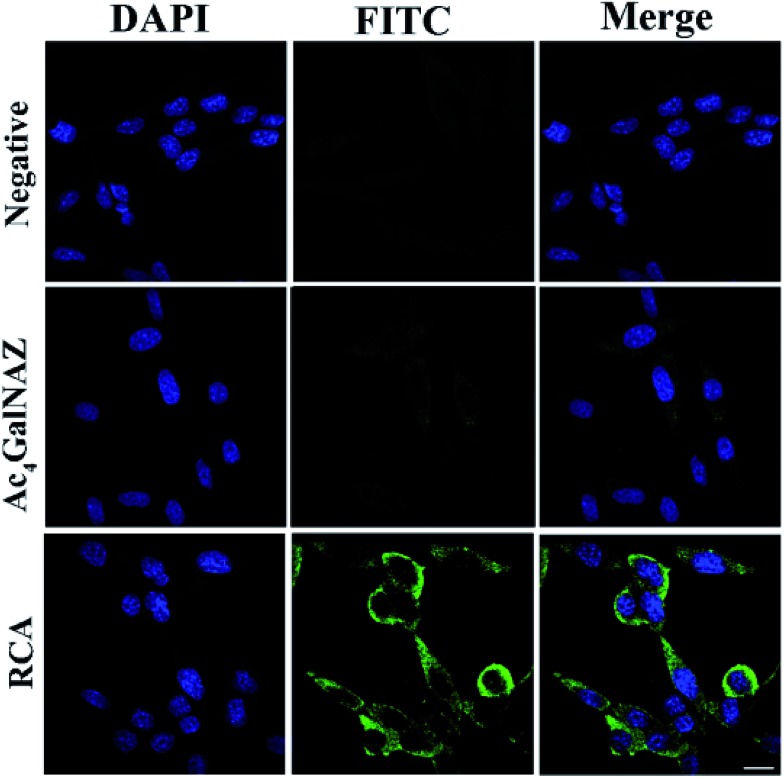
Visualization of protein glycosylation on the cell surface. 4TO7 cells cultured in 8-well slides were treated without (PMT 850 V, gain 2, and offset –30) or with 5.0 μM Ac_4_GalNAz (PMT 850 V, gain 2, and offset –30), or were further subjected to RCA (PMT 700 V, gain 2, and offset –30). The labeled glycans and nuclei of cells were stained with FITC-DNA1 (green) and DAPI (blue), respectively. Scale bars: 20 μm.

Due to the relatively high fluorescence intensity of our method, we can conveniently detect biosynthetic glycans on cells by simply using a microplate reader. Fluorescence detection of the azido-sugar-labeled cells with or without (control) RCA amplification is illustrated in Fig. 2 with the error bars representing the standard deviation of three individual experiments. The cells were cultured with 0, 0.5 or 5.0 μM Ac_4_GalNAz for 3 days. As expected, the results show that the RCA reaction can significantly enhance fluorescence intensity. Although a higher azido-sugar concentration would produce a higher fluorescence intensity, 5.0 μM unnatural sugar was selected for the cell incubation in our experiment because the fluorescence intensity at this concentration fully meets the requirements for visualization (Fig. 3) and fluorescence detection in this study. It is observed, by using the RCA technique, that we can use such a small quantity of unnatural sugar to produce high-resolution cell images and investigate the biosynthetic glycans on cells by simply using a microplate reader. The optimization of other experimental conditions, including the concentrations of alkyne-functionalized DNA S1 and FITC-DNA1, and a comparison of different catalysts, are shown in Fig. S1.†

From Fig. 2 we can also found that the amplification effect of RCA is about 21 times that of cells treated with azido-sugar but without the RCA process, in which alkyne-functionalized DNA S1 was hybridized with FITC-DNA2. It has been reported that RCA is an isothermal DNA amplification procedure and can generate a linearly concatenated DNA molecule containing up to 1000 complementary copies of the circular DNA in 1 h.^37^ The RCA reaction initiated by alkyne functionalized DNA S1 and padlock DNA was analyzed *via* electrophoresis, as shown in Fig. S2.† According to the result of agarose gel electrophoresis, the RCA reaction proceeded successfully in solution. Compared with DNA RCA in solution, the DNA RCA method in cell fluorescence imaging is performed in a heterogeneous system and the environment of cells is quite complex. Owing to these reasons, the amplification effect is not as enormous as DNA RCA performed in buffer solution. However, there is still an obvious amplification of fluorescence intensity for both confocal images (Fig. 3) and data obtained from a microplate reader (Fig. 2). Thus, DNA RCA can be effectively performed in such a complex biological environment. In addition, we also tried to directly use longer concatenated alkyne-DNA (S2) from commercial synthesis, which has four repeated FITC-DNA1 binding units (Fig. S3†), or the DNA product of RCA reaction in solution for the click reaction (data not shown), but the staining results showed that this straightforward method did not work well. This may be caused by steric effects and high charge density of the RCA product, which is repulsed by the negative charge on the cell surface. In addition to the study on fixed cells, we also tried to investigate the feasibility of this RCA method on live cells using a flow cytometric assay (Fig. S4†), and the results showed that this method was also applicable for the glycan analysis on live cells.

The fluorescence images for metabolically glycan-labeled 4TO7 cells were investigated. The cell nuclei were stained with DAPI. From the results shown in Fig. 3, virtually non-existent fluorescence was observed in the azido-sugar-untreated cells. Also, relatively weak fluorescence was observed in the azido-sugar-treated cells without the RCA process, even under a high voltage photoelectric multiplication tube (PMT) of 850 V. In the latter case, FITC-DNA2 was simply hybridized with alkyne-functionalized DNA S1 bound to azido-sugar-labeled glycans on the cell surface but there was no signal amplification. The results suggest that azido-sugar-containing glycans are displayed on the cell surface but not at a high enough level to obtain a high quality image under the present experimental conditions. However, if RCA and hybridization with FITC-DNA1 processes were sequentially carried out after metabolic glycan labeling, an intense fluorescent staining signal was consistently observed under a relatively low voltage of PMT of 700 V, which indicated that the metabolically labelled glycans on the cell surface can be successfully detected by signal amplification using the RCA reaction. A similar phenomenon was also observed in B16 cells (shown in Fig. S5†). It should be noted that, unlike many RCA reactions, which show bright dots for RCA products,^31^,^33^ carbohydrate metabolism can change all corresponding carbohydrates on a cell surface to the unnatural sugars without discrimination. Thus, a large amount of azido sugars were labeled on the cell surface. Therefore, it is impossible to see bright dots on the cell surface in the confocal cell images of our RCA products.

### Glycan metabolic labelling of different cells with different azido-sugars

**Fig. 4:**
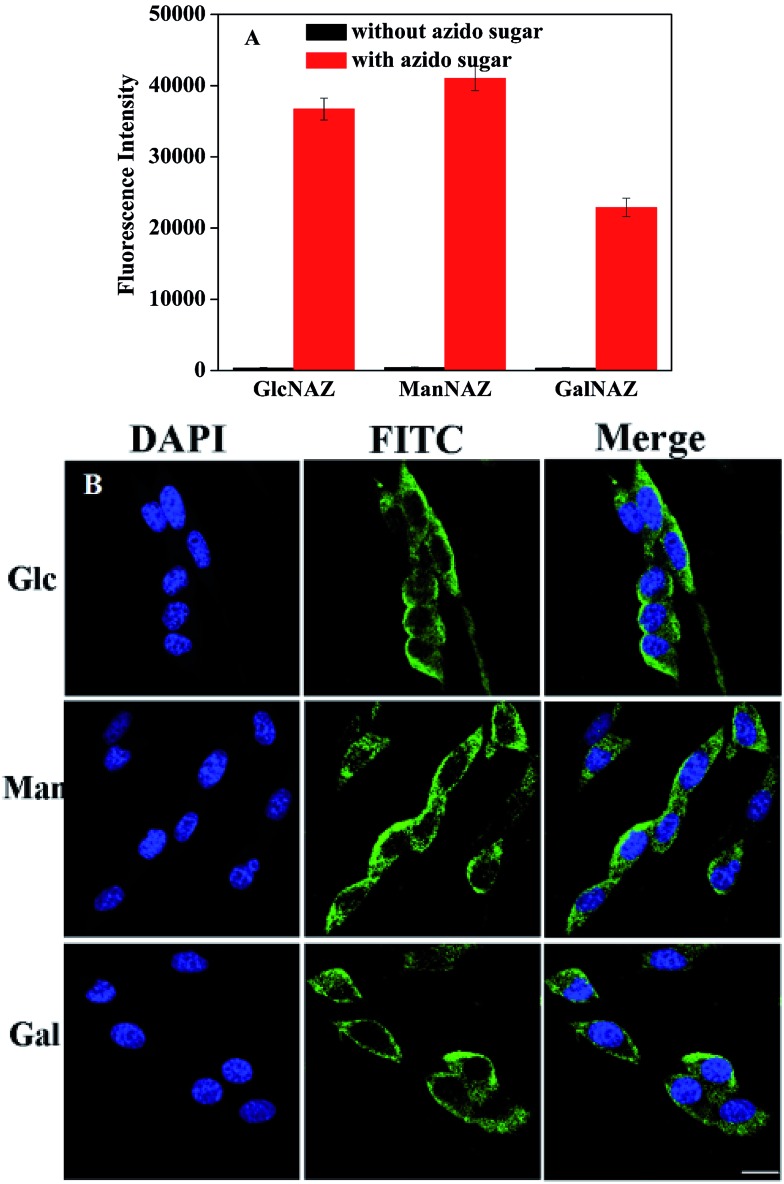
Fluorescence detection of 4TO7 cells treated with Ac_4_GlcNAz (Glc), Ac_4_ManNAz (Man) or Ac_4_GalNAz (Gal). (A) A comparison of fluorescence intensities upon incubation with 0 and 5.0 μM of different unnatural monosaccharides. (B) A comparison of fluorescence images upon treatment with 5.0 μM different unnatural monosaccharides (PMT 700 V, gain 2, and offset –30). Scale bars: 20 μm.

It has been reported that different types of unnatural monosaccharide residues can be incorporated into glycoconjugates through the cells' own biosynthetic machinery. Ac_4_ManNAz, Ac_4_GlcNAz and Ac_4_GalNAz are the most common azido-sugars used in metabolic glycan labeling. In the sugar metabolic pathway, they can convert into the corresponding azido sialic acids (SiaNAz), GalNAz and GlcNAz, respectively.^8^,^15^,^25^ Fluorescence analyses for the glycan biosynthesis of 4TO7 cells (Fig. 4A) and B16 cells (Fig. S6†) were carried out using these three per-*O*-acetylated azido-sugars. The results show that the same cell line treated with different azido-sugars show very different fluorescence intensities, and different cells treated with the same azido-sugar also show different fluorescence intensities. This phenomenon suggests that the involvement of azido-sugars in glycan synthesis is not random and the glycan type is cell type dependent. Thus, using this method we can trace the variation in carbohydrate levels at different stages of the physiological process. These phenomena can be directly observed in the fluorescence images shown in Fig. 4B.

### Design for RCA and FRET-based imaging of protein-specific glycan

Although the DNA RCA-assisted metabolic labelling method can detect glycan synthesis on cells with high sensitivity, it fails to distinguish the sugar chains of a specific glycoconjugate from all glycans. To overcome this shortcoming, the FRET technique has been used in several recent studies for imaging of protein-specific glycoforms.

**Scheme 2:**
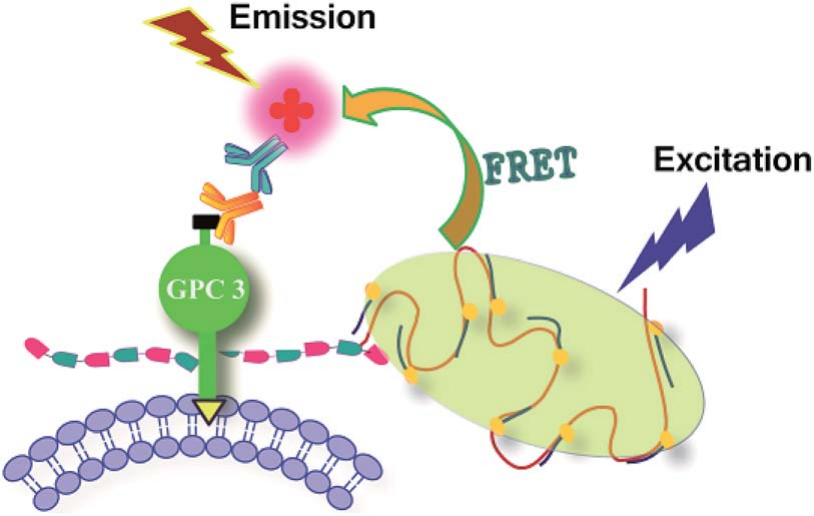
Schematic of an intramolecular FRET strategy for imaging glycans on a specific glycosylated protein. Glycan-conjugated FITC acts as a FRET donor and TRITC-conjugated anti-HA antibody acts as a FRET acceptor.

For example, Haga *et al.* used trans-membrane FRET to visualize specific protein glycoforms.^27^ Chen's group reported a cis-membrane FRET-based method for protein-specific imaging of cell-surface glycans.^35^ Ju's group developed a FRET method for simultaneous imaging of two types of monosaccharides on a specific protein by single near-infrared excitation.^38^ Here, we combined FRET with our DNA rolling circle amplified metabolic glycan labeling technique to investigate its applicability for imaging protein-specific glycans. In this study, glypican-3 (GPC3) was used as a model molecule to study glycosylation of a specific protein in living cells. GPC3 is an important cell surface proteoglycan, which has two potential heparan sulfate attachment sites^39^ and is a specific biomarker of several common cancers, in particular hepatocellular carcinoma.^40^ As shown in Scheme 2, 293T cells were transfected with the expression vector of HA-tagged GPC3. After metabolic glycan labeling, RCA amplification and hybridization with FITC-DNA1, the glycans were stained with a significant amount of FITC molecules. On the other hand, an anti-HA antibody and a TRITC-conjugated secondary antibody were successively introduced to stain the core protein of GPC3 with TRITC. Because the HA epitope is fused to the N-terminal of the core protein, the glycans and HA-tag were located on the same protein and even the same side of the cell membrane. The closely associated two subunits can produce a strong FRET-induced fluorescence signal,^41^ which facilitates investigating the influence of glycosylation on the functions of cell surface proteins. It should be noted that technically this method is not only limited to GPC3-like proteoglycans, but that other glycoconjugates such as glycoproteins and glycolipids can also be investigated by choosing appropriate azido-sugars and antibodies.

### FRET imaging of 293T cells transfected with HA-tagged GPC3

**Fig. 5:**
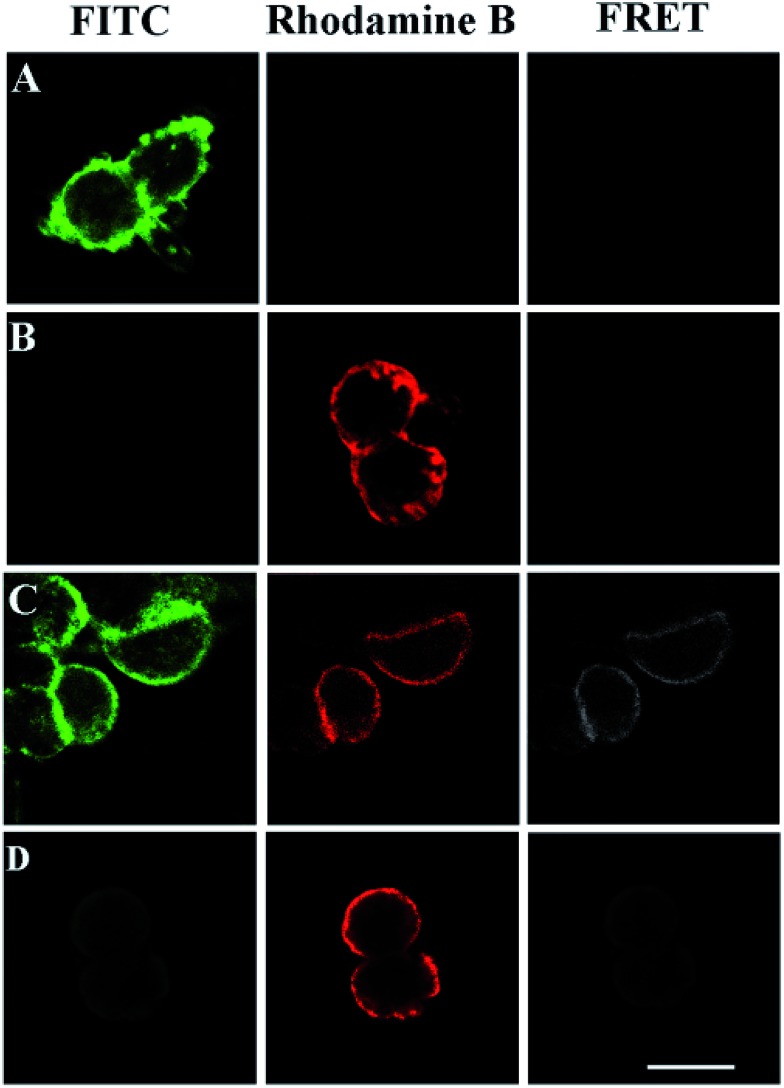
Confocal images of 293T cells. (A) The cells were treated with Ac4GalNAz followed by a click reaction with alkyne-functionalized DNA and RCA (PMT 700 V, gain 2, and offset –30); (B) the cells were transfected with HA-tagged GPC3 and stained by an anti-HA antibody and a TRITC-conjugated secondary antibody (PMT 700 V, gain 2, and offset –30); (C) both processes (A) and (B) were performed (PMT 700 V, gain 2, and offset –30); (D) similar to process (C) but without RCA (PMT 850 V, gain 2, and offset –30). Scale bars: 20 μm.

FRET phenomena between the FITC-labeled glycans and the TRITC-stained HA on the same GPC3 molecule were investigated by confocal microscopy (excitation and emission spectra for FRET pairs were investigated in Fig. S7†). As shown in Fig. 5A and B, FRET was not observed in the cells only treated with azido-sugar followed by RCA, or transfected with HA-tagged GPC3 followed by TRITC-conjugated antibody staining, which only showed FITC or TRITC fluorescence signals at the rim of the cells. Once the cells were treated with azido-sugar and transfected with HA-tagged GPC3, the cell surface could be stained by both the RCA process and anti-HA antibody (Fig. 5C). Notably, in addition to FITC staining for glycans on the surfaces of all cells and TRITC staining for GPC3 core protein on the surfaces of GPC3-expressed cells, a significant FRET-induced fluorescence on the surface of GPC3-expressed cells was observed due to the relatively near distance between the glycans and HA epitope of GPC3 molecules. The strong FITC fluorescence intensity due to RCA can induce an intense FRET signal (Fig. 5C). However, without RCA reaction, the FITC fluorescence was too weak to induce a significant FRET signal, especially in the case of reducing the amount of azido sugar (Fig. 5D) (a zoomed-out image is shown in Fig. S8†). Thus, by combining confocal FRET microscopy with an RCA reaction, researchers can conveniently and clearly image glycosylation of a specific protein. Compared with previous work, which developed a strategy for information liberation of protein specific glycosylation *via* an exonuclease III-aided recycling,^42^ our work has two obvious advantages. First, unlike the RCA process, the reported work relies on the recycling “hybridization and cleavage” process of the protein probe with other adjacent glycan probes. Therefore, the amplification effect is restricted to the number of glycans on specific proteins. However, the number of glycans is uncertain for different glycoproteins. Second, although this work demonstrates a powerful homogeneous quantification tool for research of glycosylation, the reported dual-color confocal fluorescence imaging produced a signal-off image after the scission of Exo III, while our FRET signal can be enhanced significantly after RCA, which is more suitable for fluorescence image study.

### Verification of intramolecular FRET on the same GPC3

**Fig. 6:**
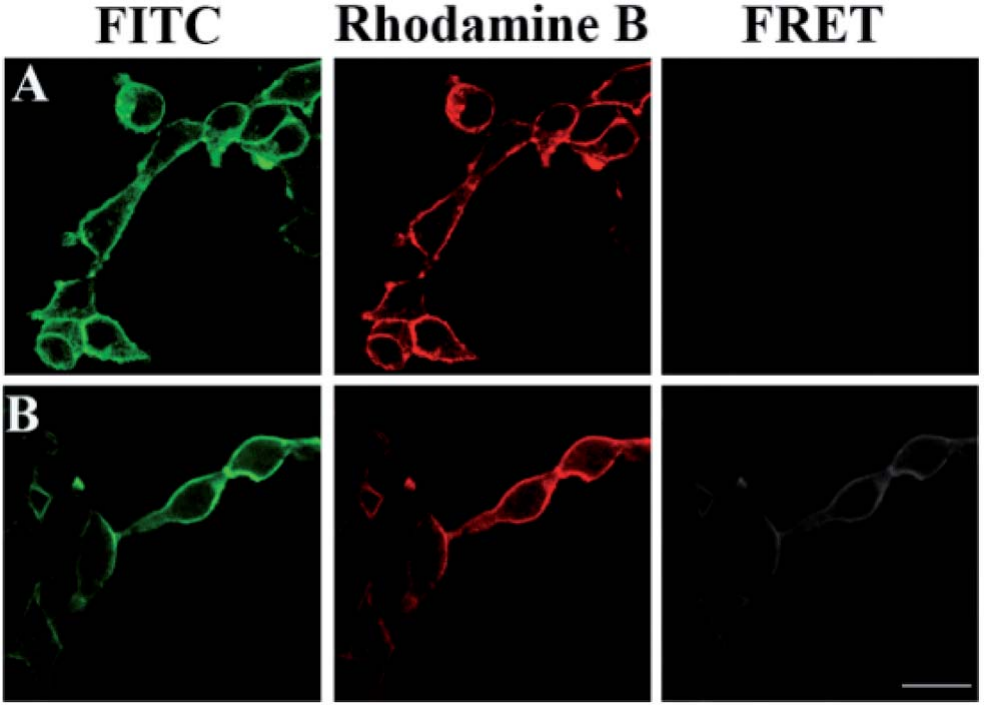
Verification of intramolecular FRET (PMT 700 V, gain 2, and offset –30). (A) Cells were transfected with HA-tagged mutant GPC3ΔGAG without glycosylation sites; (B) cells were transfected with HA-tagged wild-type GPC3. Scale bars: 20 μm.

To further verify the FRET signals come from intermolecular interactions on the same molecule, we compared fluorescence images of 293T cells transfected with wild-type GPC3 and its glycan deletion mutant GPC3ΔGAG in Fig. 6. As described above, HA-tagged wild-type GPC3 showed clear FRET images (Fig. 6A). However, because GPC3ΔGAG had no sugar chains, we only observed FITC-labeled glycans and TRITC-labeled GPC3 core proteins on the cell surface, respectively, and intramolecular FRET was absent (Fig. 6B). The results suggest that the FRET signals are not from the carbohydrates on adjacent proteins but from the same molecule. Therefore, this is a quite reliable method for glycan imaging of specific proteins.

## Experimental section

### Reagents and materials

*N*-Azidoacetylgalactosamine-tetraacylated (Ac4GalNAZ), *N*-azidoacetylglucosamine-tetraacylated (Ac4GlcNAZ), *N*-azidoacetylmannosamine-tetraacylated (Ac4ManNAZ), T4 ligase, T4 ligase buffer (10×), 4% paraformaldehyde, and trypsin were purchased from Thermo Scientific (USA). Dulbecco's modification of Eagle's medium (DMEM), Roswell Park Memorial Institute 1640 (RPMI-1640) and fetal bovine serum (FBS) were provided by Hyclone (USA). Tris(3-hydroxypropyltriazolylmethyl)amine (THPTA) was purchased from Sigma-Aldrich (USA). Phi29 polymerase and phi29 polymerase buffer (10×) were obtained from Biolab (Dalian China). The deoxynucleotide mixture (dNTPs) was purchased from SBS Genetech Co., Ltd. (China). Anti-HA monoclonal antibody and rhodamine (TRITC)-conjugated goat anti-mouse IgG (H + L) were purchased from Proteinteck (USA). Sodium l-ascorbate (SA) was purchased from J&K Technology Co., Ltd. (Beijing, China). Expression vectors of hemagglutinin A (HA)-tagged GPC3 (pEFGPC3-HA) and mutant GPC3 (GPC3ΔGAG) that cannot be glycanated (pEFGPC3ΔGAG-HA) were kindly provided by Professor Jorge Filmus. DAPI (4′,6-diamidino-2-phenylindole) was purchased from Solarbio (Beijing, China). DNA sequences used in this work are listed in Table S1.† The water used throughout this work was produced by the Milli-Q water purification system (Millipore, USA).

### Cell-surface glycoprotein labeling

Mouse breast cancer 4TO7 cells were cultured in DMEM supplemented with 10% FBS, 2 mM l-glutamine and 2 mM non-essential amino acids in 5% CO_2_ at 37 °C. Mouse melanoma B16 cells were cultured in RPMI 1640 medium supplemented with 10% FBS in 5% CO_2_ at 37 °C. Stock solutions of azido sugars (10 mM) were obtained by dissolving the sugars in filter-sterilized dimethyl sulfoxide. After seeding at a density of 2000 cells in 100 μL of media with no azido-sugar or 5.0 μM of the indicated azido sugar, and incubating at 37 °C for 72 h, the cells were fixed using 30 μL paraformaldehyde (4%) for 20 min. For the click reaction, the cells were incubated with PBS containing 250 μM THPTA, 50 μM CuSO_4_, 500 μM SA, and 50 nM alkyne-functionalized DNA S1 for 2 h at room temperature. For the RCA reaction, alkyne DNA modified cells were first treated with 50 nM padlock DNA, 1× T_4_ ligase buffer and 5 U T4 ligase in PBS buffer at 22 °C for 1 h followed by treatment with PBS containing 5 U phi29 polymerase, 1× phi29 polymerase buffer and 2.0 mM dNTP at 37 °C for 1.5 h. Finally, the cells were reacted with 3.0 μM FITC-DNA1 at 37 °C for 30 min. The cells were washed three times with PBS after each step.

For the imaging experiments, the cells were cultured in 8-well Millicell EZ slides. After the above assembly process, the cell nuclei were stained for 10 min with DAPI diluted 100 times. Next, the slide was washed with PBS, sealed and examined using a laser scanning confocal microscope.

For fluorescence intensity detection using a microplate reader, the cells were cultured in a 96-well Assay Black Plate (Corning). The RCA reaction was performed as described above. After treating with 30 μL 0.5% Triton X-100 for 20 min and washing with PBS, fluorescence intensity data were collected in triplicate.

During the control experiments, the cells were cultured without azido-sugar, or cultured with azido-sugar but without the RCA reaction, which reacted with FITC-DNA2 for fluorescent labeling.

For FRET experiments, human embryonic kidney 293T cells were cultured in DMEM containing 10% FBS and 5.0 μM azido sugar for 24 h. Then, the cells were transfected with the expression vector of pEFGPC3-HA or pEFGPC3ΔGAG-HA using Lipofectamine 2000 reagent (Invitrogen).^39^ After transfecting for 5 h, the medium was changed to the normal medium containing 5.0 μM of the indicated azido sugar and then incubated in 37 °C for 48 h. The cells were fixed using 100 μL paraformaldehyde (4%) for 20 min. Next, RCA reaction and FITC-DNA1 hybridization were performed as described above. After blocking with 200 μL 5% skim milk powder for 1 h, 100 μL of anti-HA antibody diluted 200 times was added, and the sample was incubated for 1 h in the dark. Subsequently, 200-fold diluted rhodamine (TRITC)-conjugated goat anti-mouse IgG (H + L) (100 μL) was added, and the sample was incubated for 1 h in the dark. After nuclear staining and slice sealing, the slide was used for imaging experiments. The cells were washed three times with PBS after each fabrication process to remove nonspecific adsorption.

### Cell imaging

Cell fluorescence and FRET imaging were performed using a Zeiss LSM 700 laser scanning confocal microscope. FITC fluorescence was excited at 488 nm and collected from 500 to 600 nm. Rhodamine (TRITC) was excited at 555 nm and data were collected from 560 to 700 nm. FRET-induced fluorescence was excited using a 488 nm laser, and data were collected from 600 to 700 nm. Fluorescence intensity data were detected using a PerkinElmer Multiode Plate Reader (*λ*_ex_ = 488 nm, *λ*_em_ = 525 nm).

## Conclusions

The ultrasensitive detection of glycans on a cell surface is challenging due to their low concentrations and structural complexity. Herein, a novel DNA-based RCA reaction was used for imaging cell surface glycans labeled by a metabolic method. Through the amplifying effect of the DNA cycling amplification, carbohydrates on a cell surface can be imaged using a confocal microscope or simply detected using a microplate reader with considerably less azido-sugar. By combining with FRET, the glycosylation of specific glycoconjugates can be detected using specific antibodies and appropriate azido-sugars. The technique developed herein is especially favourable for studying low-abundance cell-surface glycans, and thus, single-molecule glycan imaging may be possible.

## Supplementary Material

Supplementary information

## References

[cit1] Hanson S. R., Hsu T., Weerapana E., Kishikawa K., Simon G. M., Cravatt B. F., Wong C. H. (2007). J. Am. Chem. Soc..

[cit2] Jiang H., English B. P., Hazan R. B., Wu P., Ovryn B. (2015). Angew. Chem., Int. Ed..

[cit3] Kwon S. J., Lee K. B., Solakyildirim K., Masuko S., Ly M., Zhang F., Li L., Dordick J. S., Linhardt R. J. (2012). Angew. Chem., Int. Ed..

[cit4] Sano T., Smith C. L., Cantor C. R. (1992). Science.

[cit5] Chaubard J., Krishnamurthy C., Yi W., Smith D. F., Hsieh-Wilson L. C. (2012). J. Am. Chem. Soc..

[cit6] Chen Z. H., Liu Y., Wang Y. Z., Zhao X., Li J. H. (2013). Anal. Chem..

[cit7] Chen X. J., Wang Y. Z., Zhang Y. Y., Chen Z. H., Liu Y., Li Z. L., Li J. H. (2014). Anal. Chem..

[cit8] Hang H. C., Yu C., Kato D. L., Bertozzi C. R. (2003). Proc. Natl. Acad. Sci. U. S. A..

[cit9] Conze T., Carvalho A. S., Landegren U., Almeida R., Reis C. A., David L., Söderberg O. (2010). Glycobiology.

[cit10] Pinto R., Carvalho A. S., Conze T., Magalhães A., Picco G., Burchell J. M., Taylor-Papadimitriou J., Reis C. A., Almeida R., Mandel U., Clausen H., Söderberg O., David L. (2012). J. Cell. Mol. Med..

[cit11] Ricardo S., Marcos-Silva L., Pereira D., Pinto R., Almeida R., Söderberg O., Mandel U., Clausen H., Felix A., Lunet N., David L. (2015). Mol. Oncol..

[cit12] Kayser H., Zeitler R., Kannicht C., Grunow D., Nuck R., Reutter W. (1992). J. Biol. Chem..

[cit13] Saxon E., Bertozzi C. R. (2000). Science.

[cit14] Sawa M. T., Hsu L., Itoh T., Sugiyama M. S., Hanson R. P., Vogt K., Wong C. H. (2006). Proc. Natl. Acad. Sci. U. S. A..

[cit15] Jiang H., English B. P., Hazan R. B., Wu P., Ovryn B. (2015). Angew. Chem., Int. Ed..

[cit16] Clark P. M., Dweck J. F., Mason D. E., Hart C. R., Buck S. B., Peters E. C., Agnew B. J., Hsieh-Wilson L. C. (2008). J. Am. Chem. Soc..

[cit17] Feng L., Hong S., Rong J., You Q., Dai P., Huang R., Tan Y., Hong W., Xie C., Zhao J., Chen X. (2013). J. Am. Chem. Soc..

[cit18] Zhao X., Cai L., Adogla E. A., Guan H., Lin Y., Wang Q. (2015). Bioconjugate Chem..

[cit19] Laughlin S. T., Baskin J. M., Amacher S. L., Bertozzi C. R. (2008). Science.

[cit20] Beahm B. J., Dehnert K. W., Derr N. L., Kuhn J., Eberhart J. K., Spillmann D., Amacher S. L., Bertozzi C. R. (2014). Angew. Chem., Int. Ed..

[cit21] Agarwal P., Beahm B. J., Shieh P., Bertozzi C. R. (2015). Angew. Chem., Int. Ed..

[cit22] Attreedl M., Desbois M., Kuppevelt T. H., Bülow H. E. (2012). Nat. Methods.

[cit23] Prescher J. A., Dube D. H., Bertozzi C. R. (2004). Nature.

[cit24] Duan X. R., Cai L., Lee L. A., Chen H. X., Wamg Q. (2013). Sci. China: Chem..

[cit25] Rong J., Han J., Dong L., Tan Y., Yang H., Feng L., Wang Q., Meng R., Zhao J., Wang S., Chen X. (2014). J. Am. Chem. Soc..

[cit26] Baskina J. M., Dehnerta K. W., Laughlina S. T., Amacherb S. L., Bertozzi C. R. (2010). Proc. Natl. Acad. Sci. U. S. A..

[cit27] Haga Y., Ishii K., Hibino K., Sako Y., Ito Y., Taniguchi N., Suzuki T. (2012). Nat. Commun..

[cit28] Cheng W., Yan F., Ding L., Ju H., Yin Y. (2010). Anal. Chem..

[cit29] Wang F., Lu C., Willner I. (2014). Chem. Rev..

[cit30] Xu W. D., Deng R. J., Wang L. D., Li J. H. (2014). Anal. Chem..

[cit31] Huang S., Yu C., Cheng G., Chen Y. (2012). Anal. Chem..

[cit32] Ge J., Zhang L., Liu S., Yu R., Chu X. (2014). Anal. Chem..

[cit33] Deng R., Tang L., Tian Q., Wang Y., Lin L., Li J. (2014). Angew. Chem., Int. Ed..

[cit34] Kim J. H., Jang M., Kim Y., Ahn H. J. (2015). J. Med. Chem..

[cit35] Lin W., Du Y., Zhu Y., Chen X. (2014). J. Am. Chem. Soc..

[cit36] Chen Y., Ding L., Song W., Yang M., Ju H. (2016). Chem. Sci..

[cit37] Banér J., Nilsson M., Mendel-Hartvig M., Landegren U. (1998). Nucleic Acids Res..

[cit38] Wu N., Bao L., Ding L., Ju H. X. (2016). Angew. Chem., Int. Ed..

[cit39] Gonzalez A. D., Kaya M., Shi W., Song H., Testa J. R., Penn L. Z., Filmus J. (1998). J. Cell Biol..

[cit40] Filmus J., Capurro M. (2013). FEBS J..

[cit41] Wang Y., Tang L. H., Li Z. H., Lin Y. H., Li J. H. (2014). Nat. Protoc..

[cit42] Chen Y. L., Ding L., Song W. Y., Yang M., Ju H. X. (2016). Anal. Chem..

