# Supplementary material for: Applying DNA rolling circle amplification in fluorescence imaging of cell surface glycans labeled by a metabolic method
Source: Chem Sci. 2016 Jun 14;7(9):6182–9. doi: 10.1039/c6sc02089e (PMC6024553; doi:10.1039/c6sc02089e)
Supplement: Supplementary file 1 [file SC-007-C6SC02089E-s001.pdf]

Electronic Supplementary Material (ESI) for Chemical Science.
This journal is © The Royal Society of Chemistry 2016

Applying DNA Rolling Circle Amplification in Fluorescence Imaging of Cell Surface Glycans Labeled by Metabolic Method

Xiaoru Zhang,[a] Ruijuan Li,[a] Yuanyuan Chen,[a] Shusheng Zhang,[*, b] Wenshuang Wang,[c] Fuchuan Li[*, c]

[a]Key Laboratory of Sensor Analysis of Tumor Marker, Ministry of Education, College of Chemistry and Molecular Engineering, Qingdao University of Science and Technology, Qingdao 266042, P. R. China.

[b]Shandong Provincial Key Laboratory of Detection Technology for Tumor Markers, Linyi University, Linyi 276005, P. R. China

[c]National Glycoengineering Research Center and State Key Laboratory of Microbial Technology, Shandong University, Jinan 250100, P. R. China

[*]To whom correspondence should be addressed. Prof. Shusheng Zhang: phone, +86-539-8766107; fax, +86- 539-8766107; e-mail, zhangshusheng@lyu.edu.cn

Prof. Fuchuan Li: phone, +86-531-88365165; fax, +86-531-88363002; e-mail, fuchuanli@sdu.edu.cn.

Table S1 DNA sequences used in RCA process

Name	Sequences	Description
S1	5'-CHCHTTT TTT TTT TTT GGT GTT CAG TGT GTG GTG CG-3'	alkyne functionalized DNA
padlock DNA	5'-ACT GAA CAC CCT ACC TCC CAC GAG CAT CGC AAC CCG CCC TAC CCT GAG TGC GCA CCA CAC-3'	underline means complementary to S1
FITC-DNA1	5'-CGC AAC CCG CCC TAC CCT TTT TTT TTT-FITC-3'	complementary to RCA product
FITC-DNA2	5’-FITC-TTT TTT TTT CGC ACC ACA CAC TGA ACA-3’	complementary to S1
S2	5'-CHCH-TTT TTT TTT TTT GGT GTT CAG TGT GTG GTG CGG TTC GGG TGT TCA GTG TGT GGT GCG TTT GGT GTT CAG TGT GTG GTG CGG TTG TGG TGT TCA GTG TGT GGT GCG -3'	long sequence of alkyne functionalized DNA

Analyzing optimum reaction conditions

The experiments for optimizing reaction conditions were performed by using 96-well plates. The relative fluorescence intensities for different concentrations of alkyne-functionalized DNA S1 were investigated as shown in **Fig. S1A**. Considering the experiment effect and saving the materials, 50 nM alkyne-functionalized DNA S1 was used in the following studies. Next, since the RCA product could be hybridized with high quantities of fluorescent detection probes to give highly sensitive in situ fluorescence image, the concentrations of FITC-DNA1 should be investigated. As shown in **Fig. S1B**, with increasing the concentration of FITC-DNA, the fluorescence intensities increase rapidly, and tend to be steady since 3.0 μM. So 3.0 μM FITC-DNA1 was used as an optimum concentration.

In addition, Cu(I)-catalyzed azide–alkyne cycloaddition can be further accelerated using the Cu(I)-stabilizing ligand. THPTA (tris[(1-hydroxypropyl-1H-1,2,3-triazol-4-yl)methyl]amine), TBTA (tris[(1-benzyl-1H-1,2,3-triazol-4-yl)methyl]amine)[1] and His (histidine)[2] are three most common used Cu(I)-stabilizing ligands, and have been used to study the catalytic performance of azide–alkyne cycloaddition reactions in this study. From the data comparison shown in **Fig. S1C**, we could see that the catalytic effect of THPTA–Cu^I and TBTA–Cu^I was much better than that of His-–Cu^I, and THPTA performs even better than TBTA under equivalent conditions. Moreover, from the images shown in **Fig. S1D**, we could see that the fluorescence of FITC was localized on cell surface when catalyzed by THPTA–Cu^I, while the whole cell was stained when catalyzed by TBTA–Cu^I. This may cause by the excellent water solubility of THPTA, while TBTA has to be dissolved in DMSO. This result is coincided with previous reports that THPTA ligand was effectively used to label cells with high efficiency while maintaining cell viability.[1,3] The THPTA ligand binds Cu(I), blocking the bioavailability of Cu(I) and ameliorating the potential toxic effects while maintaining the catalytic effectiveness in click conjugations.

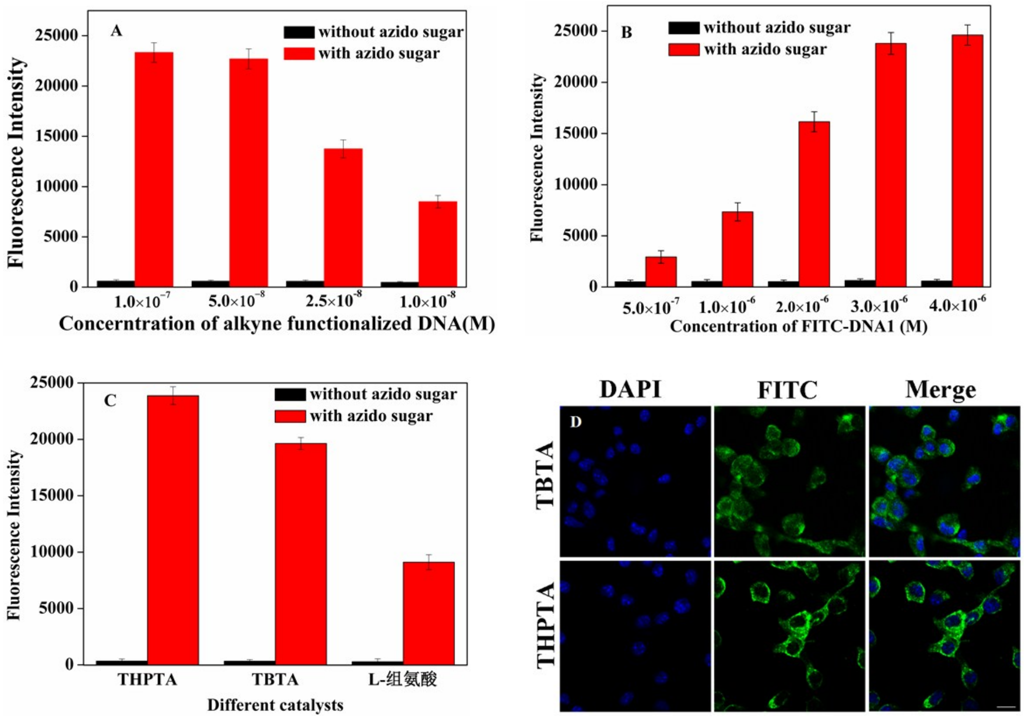

Fig. S1. Optimizing the experimental conditions. (A) Fluorescence intensity using different concentration of alkyne-functionalized DNA S1; (B) Fluorescence intensity using different concentration of FITC-DNA1; (C) Fluorescence intensity using different catalysts; and (D) Confocal fluorescence images of 4TO7 cells metabolically labeled with $Ac_4GalNAZ$, followed by a click reaction catalyzed by THPTA or TBTA, and then subjected to RCA reaction (PMT 700 V).

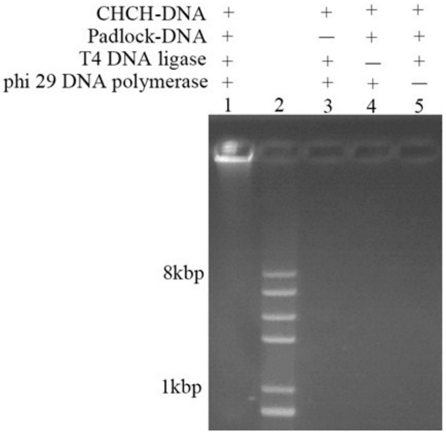

Fig. S2 Agrose gel (0.8%) electrophoresis image of RCA products. Lane 2 is the DNA size marker. Lane 1 the presence of all components in the RCA reaction. Lane 3-5 is the presence and the absence of a component in the RCA reaction were indicated by ＋and －, respectively.

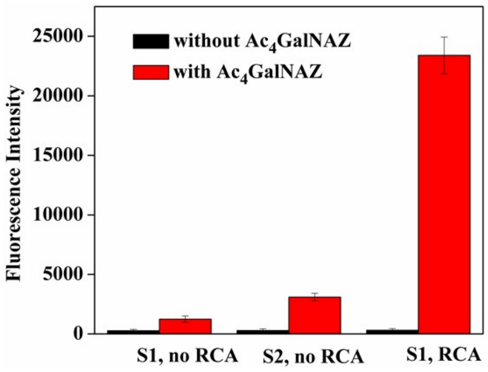

Fig. S3. Fluorescence intensity comparation of 4TO7 cells treated with alkyne-DNA S1 and longer alkyne-DNA S2.

Flow Cytometric Analysis of Labeled Cells

In order to ensure high cell viability, some operation modifications were performed during flow cytometric analysis. A mixture of 250 μM THPTA, 50 μM $CuSO_4$, 500 μM SA, and 50 nM alkyne-functionalized DNA S1in 300 μL of PBS was prereacted for 60 min on ice, and then was added into an aliquot of 3 × 10^6 293T cells labeled with 5.0 μM azido sugar as described above. After incubation for 20 min at 4°C, the cells were washed two times with PBS. For the RCA reaction, alkyne DNA modified cells were first treated with 50 nM padlock DNA, 1× T_4 ligase buffer and 5 U T4 ligase in PBS buffer (total volume is 300 μL) at 22 °C for 20 min followed by treated with PBS containing 5 U phi 29 polymerase, 1× phi 29 buffer and 2.0 mM dNTP (total volume is 300 μL) at 37 °C for 50 min. Finally, the cells were treated with 210 μL PBS containing 90 μL FITC-DNA1 (3.0 μM) at room temperature for 15 minutes. The cells were washed three times and suspended in 300 μL PBS, then immediately analyzed via flow cytometry. Trypan blue staining showed that the survival rate of the cells was about 70%.

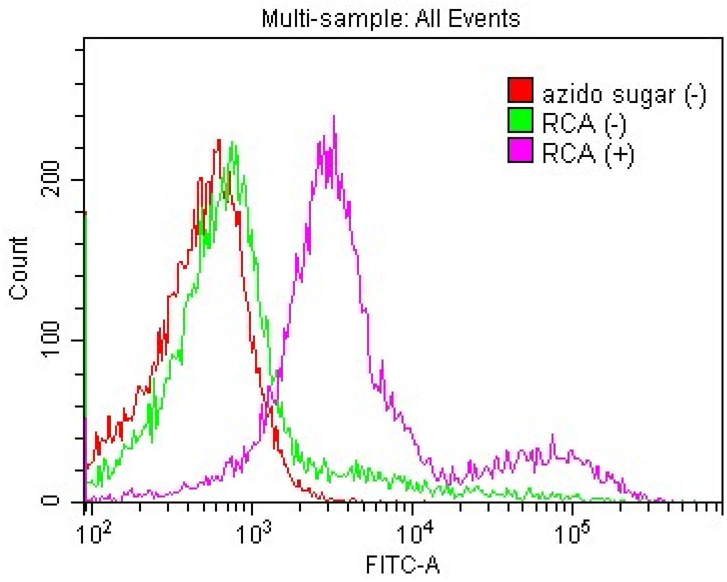

Fig. S4. Flow cytometric analysis of live 293T cells.

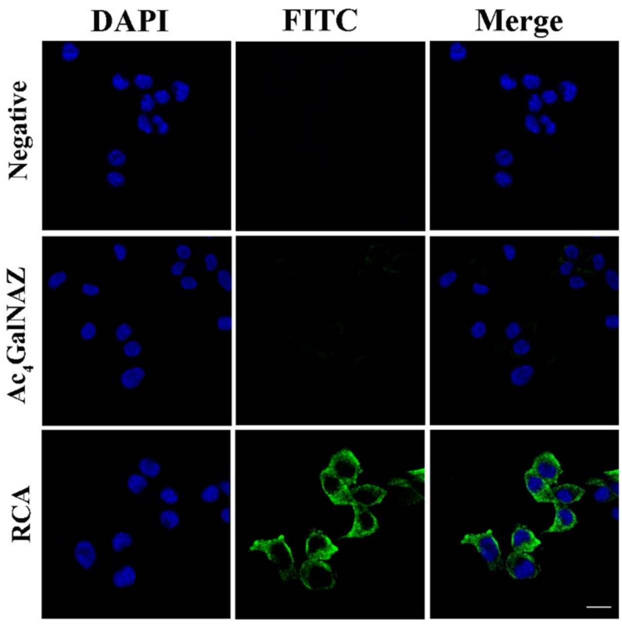

Fig. S5. Visualization of protein glycosylation (green) on the B16 cell surface. B16 cells cultured in 8-well slides were treated without or with Ac_4GalNAz, or further subjected to the RCA reaction (PMT 700 V). The nuclei were stained with DAPI (blue). Scale bars: 20 μm.

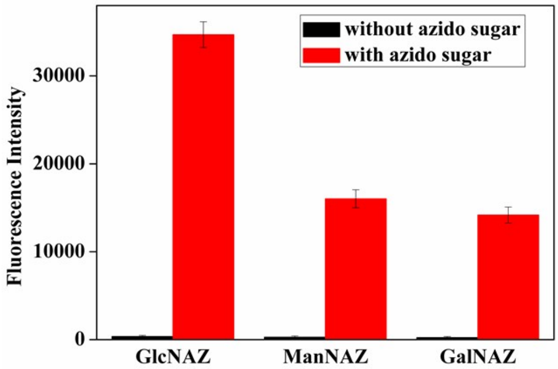

Fig. S6. Fluorescence detection for B16 cells treated with different types of unnatural monosaccharides residues.

Excitation and emission spectra for FRET pairs.

Two dyes were carefully selected for efficient fluorescence resonance energy transfer. [4,5,6] To identify candidate FRET pairs, absorption and fluorescence spectra were recorded as shown in Figure S4. We used FITC and TRITC because the FITC emission spectrum can overlap with the TRITC excitation spectrum. Furthermore, a well-separated excitation spectrum for FITC and TRITC was observed; thus, direct excitation of TRITC was minimized with FITC excitation. These conditions ensure the FRET process in this work.

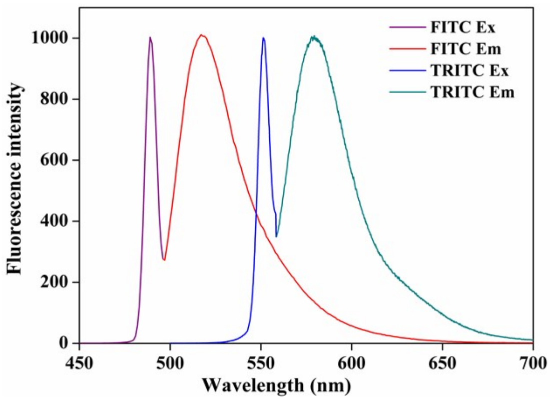

Fig. S7. Excitation and emission spectra for the FRET pairs. FITC 488 acted as a donor, and TRITC 550 acted as an acceptor. The excitation and emission spectra were measured using an F4500 fluorometer. The intensities were normalized to comparable levels.

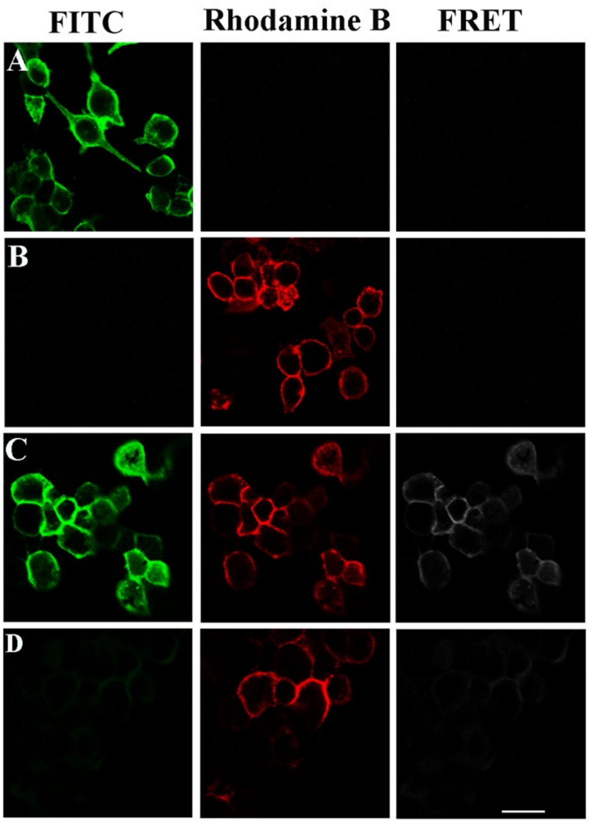

Fig. S8. Zoomed-out view for FRET experiments. (A) The 293T cells were treated with $Ac_4GalNAz$ followed by a click reaction with alkyne-functionalized DNA and RCA (PMT 700 V). (B) The cells were transfected with the expression plasmid of HA–tagged GPC3, and then stained by the anti-HA antibody and rhodamine (TRITC)-conjugated secondary antibody (PMT 700 V). (C) Both processes of (A) and (B) were performed (PMT 700 V). (D) Similar to process (C) but without RCA (PMT 850 V). Scale bars: 20 μm.

References

[1] C. Besanceney-Webler, H. Jiang, T. Zheng, L. Feng, D. S. del Amo, W. Wang, L. M. Klivansky, F. L. Marlow, Y. Liu, and P. Wu, *Angew. Chem. Int. Ed*. **2011**, 50, 8051 –8056.

[2] D. C. Kennedy, C. S. McKay, M. C. B. Legault, D. C. Danielson, J. A. Blake, A. F. Pegoraro, A. Stolow, Z. Mester, J. P. Pezacki， *J. Am. Chem. Soc.* **2011**, 133, 17993 – 18001.

[3] V. Hong, N. F. Steinmetz, M. Manchester, M. G. Finn, *Bioconjugate Chemistry*, **2010**, 21, 1912-1916.

[4] X. Wu, Z. Li, L. Yang, J. Han, S. Han, *Chem. Sci*. **2013**, 4, 460 - 467

[6] H. Kojima, N. Spataru, Y. Kawata, S. Yano, I. Vartires, *J. Phys. Chem. B***1998**, 102, 9981 - 9984.

[6] J. Zhang, Y. Lv, B. Wang, S. Zhao, M. Tan, G. Lv, X. Ma, *Mol. Pharmaceutics* **2015**, 12, 695 – 705
